# Molecular Analysis of 55 Spanish Patients with Acute Intermittent Porphyria

**DOI:** 10.3390/genes11080924

**Published:** 2020-08-12

**Authors:** María-José Morán-Jiménez, María-José Borrero-Corte, Fátima Jara-Rubio, Inmaculada García-Pastor, Silvia Díaz-Díaz, Francisco-Javier Castelbón-Fernandez, Rafael Enríquez-de-Salamanca, Manuel Méndez

**Affiliations:** 1 Instituto de Investigación Sanitaria Hospital 12 de Octubre (imas12), Fundación para la Investigación Biomédica del Hospital 12 de Octubre, Centro de Investigación, Avenida de Córdoba s/n, 28041 Madrid, Spain; moranjimenez@h12o.es (M.-J.M.-J.); mjose.borrero@hotmail.com (M.-J.B.-C.); fatimatalaveranet@gmail.com (F.J.-R.); macu.garcia.pastor@gmail.com (I.G.-P.); salamanca@med.ucm.es (R.E.-d.-S.); 2 Servicio de Análisis Clínicos, Hospital 12 de Octubre, 28041 Madrid, Spain; sddiaz@salud.madrid.org; 3 Servicio de Medicina Interna, Hospital 12 de Octubre, 28041 Madrid, Spain; fjcastelbon@yahoo.es

**Keywords:** porphyria, acute intermittent porphyria, hydroxymethylbilane synthase, porphobilinogen deaminase, mutation analysis, splicing defect, prokaryotic expression

## Abstract

Acute intermittent porphyria (AIP) results from a decreased activity of hepatic hydroxymethylbilane synthase (HMBS), the third enzyme in the heme biosynthetic pathway. AIP is an autosomal dominant disorder with incomplete penetrance, characterized by acute neurovisceral attacks precipitated by several factors that induce the hepatic 5-aminolevulinic acid synthase, the first enzyme in the heme biosynthesis. Thus, a deficiency in HMBS activity results in an overproduction of porphyrin precursors and the clinical manifestation of the disease. Early diagnosis and counselling are essential to prevent attacks, and mutation analysis is the most accurate method to identify asymptomatic carriers in AIP families. In the present study, we have investigated the molecular defects in 55 unrelated Spanish patients with AIP, identifying 32 *HMBS* gene mutations, of which six were novel and ten were found in more than one patient. The novel mutations included a missense, an insertion, two deletions, and two splice site variants. Prokaryotic expression studies demonstrated the detrimental effect for the missense mutation, whereas reverse transcription-PCR and sequencing showed aberrant splicing caused by each splice site mutation. These results will allow for an accurate diagnosis of carriers of the disease in these families. Furthermore, they increase the knowledge about the molecular heterogeneity of AIP in Spain.

## 1. Introduction

Acute intermittent porphyria (AIP; OMIM 176000) is the most common acute hepatic porphyria, and is inherited as an autosomal dominant trait with incomplete penetrance [1]. AIP results from a partial deficiency of hydroxymethylbilane synthase (HMBS; EC 4.3.1.8), also named porphobilinogen deaminase (PBGD), the third enzyme in the heme biosynthetic pathway. Human HMBS is a cytoplasmic monomeric enzyme that catalyzes the polymerization of four porphobilinogen (PBG) molecules to form a linear tetrapyrrole (hydroxymethylbilane), using the dipyrromethene cofactor [2,3].

The disease is more frequent in women than in men, and usually manifests during or after puberty, with recurrent acute attacks with neurovisceral manifestations, including abdominal pain, vomiting, constipation, hypertension, tachycardia, peripheral neuropathy, and psychiatric disturbances [1]. Acute attacks are triggered by several factors, including steroid hormones, certain drugs, alcohol, and fasting, which induce hepatic 5-aminolevulinic acid synthase (ALAS1), the first and rate-limiting enzyme in the heme biosynthesis [1]. The increase in ALAS1 activity enhances the production of porphyrin precursors, 5-aminolevulinic acid (ALA), and PBG [1,4]. Then, a deficiency in the activity of hepatic HMBS causes an overproduction and massive urinary excretion of ALA and PBG, along with clinical manifestations [1,4]. Therefore, early diagnosis and counselling regarding precipitating factors is essential to manage and prevent acute attacks in symptomatic patients and in latent heterozygous relatives. During an acute attack of AIP, patients excrete high amounts of precursors, and the urine is positive for the qualitative detection of PBG by means of the Hoesch test [5]. In urine, PBG spontaneously polymerizes to uroporphyrin and, therefore, patients with AIP excrete an increased amount of uroporphyrin [6].

The human *HMBS* gene has been mapped to the chromosomal region 11q24.1-q24.2, spanning a genomic interval of 10 kb. It contains 15 exons and produces two mRNA transcribed from different promoters, the housekeeping (in 5′ end untranslated region) and the erythroid-specific (in exon 1), which encode the ubiquitous and erythroid isoforms, respectively [7,8,9]. The housekeeping mRNA contains exon 1 joined to exons 3–15 with the translation start codon in exon 1, and encodes an enzyme that contains 361 amino acids (~42 KD), whereas the erythroid-specific mRNA contains exons 2–15 with the translation start site located within exon 3, and it encodes an enzyme of 344 amino acids (~40 KD) [10,11]. The crystal structure of the ubiquitous human HMBS reveals that it consists of three distinct α/β domains of a similar size, with the active site between domains 1 and 2 [2,3,12].

Two subtypes of AIP have been described, a classical and a non-erythroid form [1,13,14]. In the classical AIP, the HMBS activity is reduced to about 50% of what is normal in all of the tissues, due to a mutation that affects both isoforms of the enzyme. In the non-erythroid variant AIP (2–5% of cases), the mutation only affects the housekeeping isoform and patients have a normal HMBS activity in the erythrocytes. In asymptomatic (latent) AIP patients, the levels of porphyrin precursors in urine may be normal [1]. The measurement of the erythrocyte HMBS activity can be used to identify these individuals, however this is limited to classical AIP. Moreover, there is a significant overlap in the activity values between the heterozygote and normal individuals [7]. Therefore, when a disease-causing mutation has been identified in a proband, a molecular analysis is the most accurate method to identify asymptomatic heterozygotes in the family [7,15]. To date, over 400 disease-causing mutations have been described in the *HMBS* gene identified in AIP patients (Human Gene Mutation Database (HGMD), http://www.hgmd.cf.ac.uk/ac/index.php).

## 2. Materials and Methods

### 2.1. Patients and Biochemical Determinations

In this report, we studied 55 unrelated Spanish patients with AIP—47 females and 8 males. The patients were diagnosed and followed up clinically at the Hospital 12 de Octubre (Madrid, Spain). They presented a clinical history of at least one acute attack associated with an increased excretion of ALA, PBG, and porphyrins in their urine. The age at the first attack ranged from 16 to 62 years. In these patients, the erythrocyte HMBS activity was measured as previously described [16]. The HMBS activity was measured using PBG as a substrate, and the product, spontaneously converted to uroporphyrinogen I, was oxidized to uroporphyrin I by light exposition and was then fluorimetrically quantified.

All of the patients gave informed consent prior to their inclusion in the study. The study was conducted in accordance with the Declaration of Helsinki and the study protocol was approved by the Ethical Committee of the Hospital 12 de Octubre (2013–0034).

### 2.2. DNA Analysis

Blood samples were collected in tubes containing EDTA, and genomic DNA was extracted using the NZY blood gDNA isolation kit (NZYtech, Lisboa, Portugal). The *HMBS* gene was PCR amplified and sequenced using the primers described [16], but without the GC clamp. All 15 exons with their flanking intron regions were amplified in five fragments, as previously described [17]. All of the mutations were confirmed on a second DNA sample. The nucleotides were numbered according to the cDNA sequence for the housekeeping isoform of *HMBS* (GenBank Accession Number NM_000190), in which the A of the ATG initiation codon was numbered as 1.

### 2.3. RNA Analysis

The effect of two newly identified intronic mutations (c.161–3C > G and c.651+3A > T) on the mRNA splicing was studied by reverse transcription-PCR (RT-PCR) and sequencing. Leukocytes were isolated from EDTA-anticoagulated blood using Ficoll-Paque Plus (Amersham Biosciences, Uppsala, Sweden), and total the RNA was extracted using TRIzol Reagent (Invitrogen, Carlsbad, CA, USA). Reverse transcription was carried out with an oligo (dT) primer and eAMV reverse transcriptase (Sigma-Aldrich, Inc. St. Louis, MO, USA). Subsequently, the *HMBS* cDNA was amplified, and the RT-PCR products were run on an agarose gel and sequenced. For the c.161–3C > G mutation, the sense primer was 5′-ATTCGCGTGGGTACCCGCAAGAGC-3′ and the antisense primer was 5′-GTCCTTCAAGGAGTGAACAACC-3′, whereas for the c.651+3A > T mutation, the sense and antisense primers were 5′-ATTCGCGTGGGTACCCGCAAGAGC-3′ and 5′-TAGGCACTGGACAGCAGCAACCCA-3′, respectively. When more than one band was observed, each product was cut out of the gel, purified using the GFX PCR DNA and gel Band Purification kit (GE Healthcare, Madrid, Spain), and sequenced separately.

### 2.4. Prokaryotic Expression and Characterization of the Novel HMBS Missense Mutation

**Figure 1:**
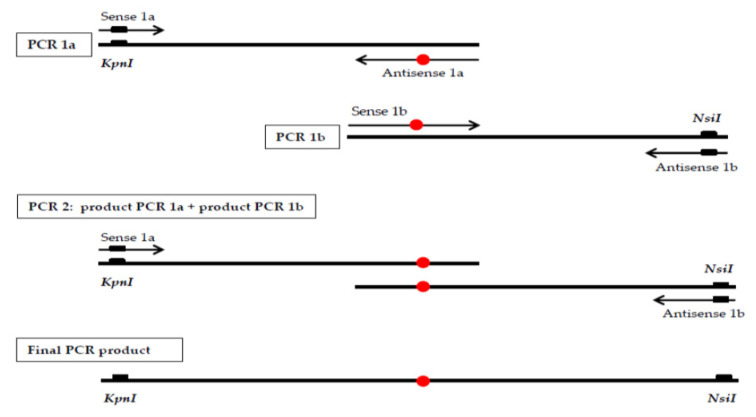
Two PCR steps to generate a fragment containing the c.294G > T mutation, marked with a red circle.

The novel missense mutation was expressed in *Escherichia coli* strain JM109 (Promega Corporation. Madison, WI, USA) using the pKK223-3 expression vector (Pharmacia Biotech Inc., Piscataway, NJ, USA). The pKK-HMBS-wt plasmid, in which the cDNA encoding the human wild-type housekeeping *HMBS,* was cloned into the *EcoRI-HindIII* sites of the pKK223-3 and used as the normal construct [17]. The missense mutation c.294G > T (p.K98N) was introduced into the pKK-HMBS-wt by PCR-based site-directed mutagenesis [18]. To generate the mutant construct pKK-HMBS-K98N, a DNA fragment containing the nucleotide substitution with flanking *KpnI* and *NsiI* restriction sites for cloning was generated in two PCR steps (Figure 1). First, two overlapping PCR fragments containing the mutation and a restriction site were generated separately, using pKK-HMBS-wt plasmid as the template. The primer pairs for each PCR were (a) sense 5′-ATTCGCGTGGGTACCCGCAAGAGC-3′ (the restriction site for *KpnI* is underlined) and antisense 5′-CACAGTGGGCAGGTC**a**TTCAAGGAGTGAAC-3′, and (b) sense 5′-GTTCACTCCTTGAA**t**GACCTGCCCACTGTG-3′ and antisense 5′-CCACAGCATACATGCATTCC-3′ (the restriction site for *NsiI* is underlined); in the mutagenesis primers, the mutated base is in bold lower case letters. After purification, both PCR fragments were used together as templates in a second PCR step using the primers with restriction sites. Then, the final PCR product was digested with the restriction enzymes *KpnI* and *NsiI* (New England Biolabs, Beverly, MA, USA), and purified using the GFX PCR DNA and gel Band Purification kit (GE Healthcare, Little Chalfont, UK). After purification, this fragment was ligated into the pKK-HMBS-wt, which had been digested with the same enzymes, and the resulting plasmid was transformed in *Escherichia coli* strain JM109. The integrity of the expression construct was confirmed by automated sequencing.

Bacterial clones, containing either the pKK223–3 vector or any of the pKK-HMBS expression constructs, were grown to logarithmic phase and induced with 5 mM isopropylthiogalactoside (IPTG) for 3 h. The cells were harvested by centrifugation and washed twice with PBS. The cell pellets were resuspended in 200 µL of lysis buffer (100 mM Tris-HCl buffer, pH 8.0, 0.1% Triton-X 100) and disrupted by sonication. The bacterial lysates were centrifuged, and the supernatants were used as the source of the enzyme. The HMBS activity was measured as published [16]. The specific activity (SA) was calculated as nmol of uroporphyrinogen formed per hour and mg of protein. Four independent experiments were performed, and the results were averaged. The residual activity of the mutation was calculated by dividing 100 × (SA–SA (pKK223-3)) by (SA (pKK-HMBS-wt)–SA (pKK223-3)).

## 3. Results

### 3.1. Patients

**Figure 2:**
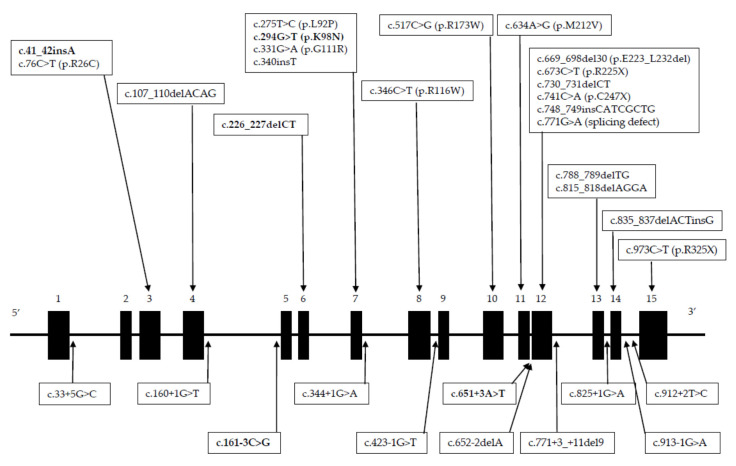
Diagram of locations of mutations in the *HMBS* gene. The black rectangles represent the exons and the lines represent the introns. Mutations found in exons and introns are shown at the top and bottom of the diagram, respectively. The novel mutations found in this study are in bold.

Most of the patients studied displayed erythrocyte HMBS activities ranging from 24% to 78% of the control value, and one patient presented a normal activity (Table 1 and Figure 2). In each patient, we identified a *HMBS* mutation in a heterozygous state. Most were localized between exons 3 to 15, except for the patient with the normal HMBS activity, which occurred in the donor site of intron 1. Then, in this series, the classical AIP represents 98% of patients.

### 3.2. Mutations Identified

**Figure 3:**
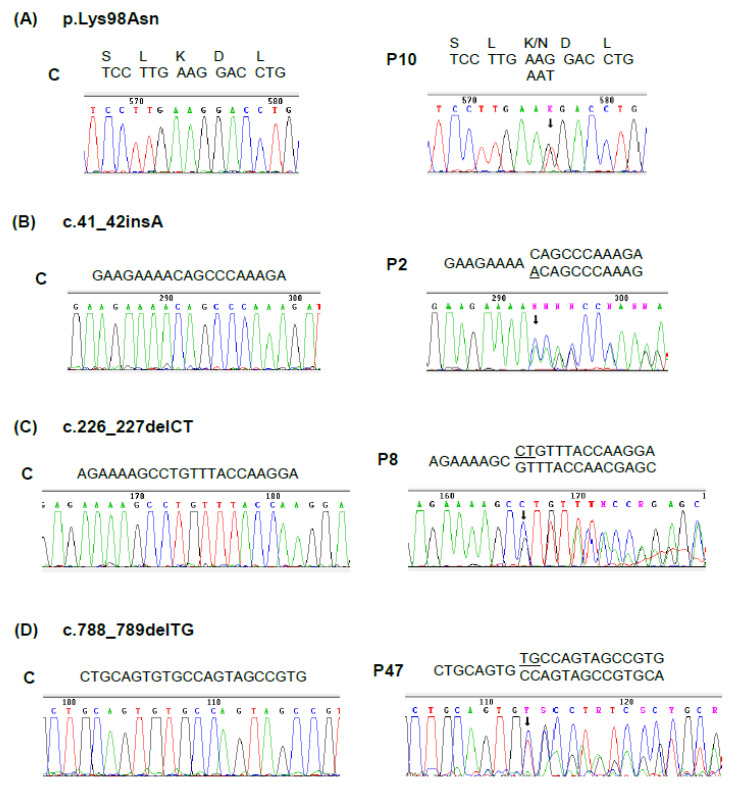
Novel exonic mutations in the *HMBS* gene. Electropherograms showing the relevant parts of the genomic sequence in a (C) control individual and in the (P) affected patients. (**A**) Missense mutation, the position of the mutated nucleotide is indicated by an arrow and the amino acid sequences are shown. (**B**) Small insertion, the arrow indicates the site of the insertion and the nucleotide inserted is underlined. (**C**,**D**): Small deletions, the arrow indicates the beginning of the deletion and the deleted nucleotides are underlined. Patients are numbered according to Table 1.

**Figure 4:**
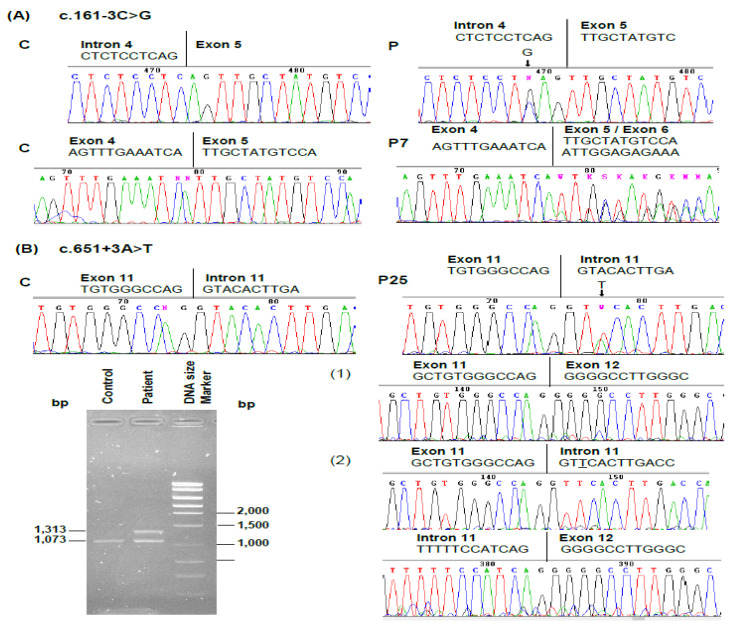
Characterization of the novel splice site mutations in the *HMBS* gene. Electropherograms showing relevant parts of the genomic and cDNA sequences in a (C) control individual and in the (P) affected patients. (**A**): Upper panel: Identification of the G to C transversion at the 3′ junction of intron 4. The mutated nucleotide is indicated by an arrow. Lower panel: partial sequence electropherograms of the RT-PCR products from a control individual and from the patient. (**B**) Upper panel: Identification of the A to T transversion at the 5′ junction of intron 11. The mutated nucleotide is indicated by an arrow. Lower panel: Agarose-gel electrophoresis of the *HMBS* RT-PCR products from a control individual and from the affected patient (left). Partial sequence electropherograms of the RT-PCR products from the patient (right), (1) a normal size product, and (2) a bigger product in which intron 11 has been retained. Patients are numbered according to Table 1.

A total of 6 novel and 26 previously reported mutations were identified in these probands (Table 1). The novel mutations included a missense, an insertion, two deletions, and two splicing defects (Figure 3 and Figure 4). Of the already known mutations, 14 were not previously reported in the Spanish population and 10 were found in more than one proband, the most frequent, identified in 11 patients as being c.669_698del30 (Table 1).

The novel missense mutation was a transversion c.294G > T (p.K98N) in exon 7. This mutation expressed in *E. coli* rendered a residual activity of less than 1% of the wild-type HMBS activity. The insertion lies in exon 3 and consists of a single adenine insertion in a track of four adenines between positions c.38 and c.41, designated as c.41_42insA. This microinsertion causes a frameshift and introduces a stop signal of translation at codon 52, in exon 4. The two deletions also caused a change in the reading frame with the introduction of a premature stop codon. These were microdeletions—a dinucleotide CT deletion of nucleotides c.226 and c.227 in exon 6 (designated c.226_227delCT), which creates a stop of translation five codons downstream in the same exon. Finally, a dinucleotide GT deletion of nucleotides c.788 and c.789 in exon 13 (designated c.788_789delGT) resulted in a stop codon at position 289 in exon 14.

Moreover, two novel splicing defects were identified. First, a C to G transversion in intron 4, at the acceptor site (position c.161-3). RT-PCR and sequencing show that this defect results in the skipping of exon 5 (Figure 4A). The second was located in intron 11 and consisted of an A to T transversion at the donor site (position c.651 + 3). In this case, RT-PCR studies have shown that the mutation results in the retention of the entire 240 nucleotides of intron 11 in the mature transcript (Figure 4B).

## 4. Discussion

In this study, we measured the erythrocyte HMBS activity and performed a mutational analysis of the *HMBS* gene in 55 unrelated Spanish patients with AIP. Among these patients, one had the non-erythroid variant of AIP. In the patients studied, 32 different mutations were identified, of which 6 were novel mutations and 10 were found in more than one patient. The most frequent mutation was c.669_698del30, which was found in 11 patients. This is a previously described mutation that is common in Spain [17,31]. Of the reported mutations, 14 were found for the first time in the Spanish population.

The novel missense mutation p.K98N affects a residue that is invariant through evolution [2,3]. Lysine 98 is part of domain 1 of HMBS and interacts with the dipyrromethene cofactor at the active site [3]. Prokaryotic expression studies revealed that this change produces a protein with little, if any, residual activity. A previously identified disease-causing mutation at the same residue (p.K98R) had a similar activity when expressed in *E coli* [3,20].

Three novel frameshift mutations were found, namely, c.41_42insA, c.226_227delCT, and c.788_789delTG, which cause premature termination codons (PTCs) in exons 4, 6, and 13, respectively. Accordingly, the mutant transcripts are most likely degraded by nonsense-mediated mRNA decay (NMD) [40,41].

Two novel intronic mutations that affect consensus splice sites were found, namely: c.161–3C > G in the acceptor site of intron 4 (IVS4–3C > G) and c.651 + 3A>T in the donor site of intron 11 (IVS11 + 3A > T). RT-PCR studies showed that both mutations cause aberrant splicing. The c.161–3C > G affects a conserved nucleotide in the intronic acceptor site sequence [42,43]. This mutation causes the deletion of the 50 nucleotides of exon 5, which causes a change in the reading frame and introduces a stop codon after nucleotide 32. In the case of mutation c.651 + 3A > T, a conserved purine nucleotide is replaced with a pyrimidine nucleotide within the splice donor site [42,43]. This mutation results in the retention of the 240 nucleotides of intron 11, an in-frame insertion that contains several stop codons. Intron 11 retention instead of exon 11 skipping is also the consequence of two other mutations at the same donor site, c.651 + 1G > C and c.651 + 2T > C [24,39]. As these splicing defects introduce PTCs, the mutant transcripts are a target for degradation by NMD [40,41].

In our series, no correlation between genotype–phenotype was observed, which is in agreement with previous studies [37,38]. Intragenic polymorphisms were studied elsewhere so as to establish a possible relation with clinical manifestation, however no association was found [37].

The identification of the mutation underlying the disease in these probands will allow for the accurate diagnosis of asymptomatic heterozygotes in their families, to provide counselling to those individuals, and to avoid precipitating factors of the disease. Furthermore, these results emphasize the molecular heterogeneity of AIP in Spain.

## Figures and Tables

**Table 1:** Biochemical data and hydroxymethylbilane synthase (*HMBS*) gene mutations identified in this study.

Patient (Sex) Age ^1^	ALA (mg/L) n.r. ≤ 7.5	PBG (mg/L) n.r. ≤ 2.5	Total Porphyrins (µg/g Creatinine) n.r. ≤ 200	HMBS Activity ^2^	Mutation ^3^	Location	Reference
P1 (F) 22	60	47	22,025	105	c.33+5G > C (splicing defect) ^4^	Intron 1	[19]
P2 (F) 33	13	21	435	48	c.41_42insA	Exon 3	This study
P3 (F) 29	114	104	1120	52	c.76C > T (p.R26C)	Exon 3	[20]
P4 (F) 26	9	12	716	33			
P5 (F) 23	9	10	364	69	c.107_110delACAG ^4^	Exon 4	[21]
P6 (F) 28	8	11	993	55	c.160 + 1G > T (splicing defect) ^4^	Intron 4	[16]
P7 (F) 45	14	39	234	62	c.161–3C > G (splicing defect)	Intron 4	This study
P8 (F) 38	n.a.	n.a.	n.a.	75	c.226_227delCT	Exon 6	This study
P9 (M) 62	n.a.	n.a.	n.a.	53	c.275T > C (p.L92P)	Exon 7	[22]
P10 (F) 34	57	151	1926	61	c.294G > T (p.K98N)	Exon 7	This study
P11 (F) 29	n.a.	n.a.	n.a.	47	c.331G > A (p.G111R)	Exon 7	[23]
P12 (F) 61	20	35	n.a.	72			
P13 (F) 32	n.a.	n.a.	n.a.	50	c.340insT	Exon 7	[24]
P14 (F) 38	73	21	23,381	57			
P15 (F) 21	37	41	1232	60			
P16 (F) 28	n.a.	n.a.	n.a.	46	c.344 + 1G > A (splicing defect) ^4^	Intron 7	[25]
P17 (F) 46	67	45	910	60	c.346C > T (p.R116W)	Exon 8	[26]
P18 (F) 19	17	47	392	48			
P19 (M) 25	n.a.	n.a.	n.a.	44			
P20 (F) 26	14	21	459	46	c.423–1G > T (splicing defect) ^4^	Intron 8	[27]
P21 (F) 34	25	50	794	44	c.517C > G (p.R173W)	Exon 10	[28]
P22 (F) 22	47	25	381	59			
P23 (F) 28	14	71	720	55			
P24 (F) 51	n.a.	n.a.	n.a.	70	c.634A > G (p.M212V)	Exon 11	[29]
P25 (F) 17	127	143	527	59	c.651 + 3A > T (splicing defect)	Intron 11	This study
P26 (F) 27	55	74	n.a.	58	c.652–2delA (splicing defect)	Intron 11	[30]
P27 (F) 43	10	5	669	71	c.669_698del30 (p.E223_L232del)	Exon 12	[31]
P28 (F) 26	30	7	316	55			
P29 (F) 36	38	156	1403	52			
P30 (F) 30	83	235	127	49			
P31 (M) 40	28	66	374	56			
P32 (F) 35	n.a.	n.a.	1578	44			
P33 (F) 56	n.a.	n.a.	n.a.	24			
P34 (F) 37	n.a.	n.a.	n.a.	54			
P35 (M) 38	n.a.	n.a.	358	58			
P36 (F) 41	10	4	908	64			
P37 (F) 62	n.a.	n.a.	n.a.	48			
P38 (F) 25	125	109	794	64	c.673C > T (p.R225X)	Exon 12	[20]
P39 (M) 56	n.a.	n.a.	n.a.	61	c.730_731delCT ^4^	Exon 12	[32]
P40 (F) 28	n.a.	n.a.	n.a.	60			
P41 (F) 49	55	103	1115	55	c.741C > A (p.C247X) ^4^	Exon 12	[24]
P42 (F) 44	31	31	524	65	c.748_749insCATCGCTG ^4^	Exon 12	[33]
P43 (M) 37	n.a.	n.a.	n.a.	45	c.771G > A (splicing defect) ^4^	Exon12	[34]
P44 (F) 57	n.a.	n.a.	n.a.	57	c.771 + 3_ +11del9 (splicing defect)	Intron 12	[17]
P45 (F) 36	21	28	801	48			
P46 (F) 20	13	26	973	55			
P47 (M) 42	41	124	608	65	c.788_789delTG	Exon 13	This study
P48 (F) 41	19	68	11,258	55	c.815_818delAGGA ^4^	Exon 13	[35]
P49 (F) 23	37	62	887	74	c.825 + 1G > A (splicing defect) ^4^	Intron 13	[36]
P50 (F) 28	n.a.	n.a.	n.a.	61			
P51 (F) 28	12	32	246	68	c.835_837delACTinsG	Exon 14	[29]
P52 (F) 39	9	13	498	60	c.912 + 2T > C (splicing defect) ^4^	Intron 14	[37]
P53 (F) 16	43	89	992	50	c.913–1G > A (splicing defect) ^4^	Intron 14	[38]
P54 (M) 38	20	23	2264	53	c.973C > T (p.R325X) ^4^	Exon 15	[39]
P55 (F) 30	38	146	1266	78			

**^1^** Disease onset (years). Urinary precursors and porphyrin values at onset. Normal range: n.r. No data available: n.a. **^2^** HMBS: erythrocyte hydroxymethylbilane synthase activity expressed as the percentage of the mean value from 50 healthy individuals (mean ± standard deviation (SD): 98.5 ± 14.2 pmol uroporphyrinogen/hr/mg hemoglobin). **^3^** The absence of these sequence deviations was confirmed in 50 unrelated healty (non-porphyric) individuals of Spanish origin. Reference sequence: GeneBank Accession number NM_000190 (*HMBS* cDNA). **^4^** Mutations previously unreported in the Spanish population.
